# Association between Frailty and Mortality, Falls, and Hospitalization among Patients with Hypertension: A Systematic Review and Meta-Analysis

**DOI:** 10.1155/2021/2690296

**Published:** 2021-01-27

**Authors:** Kaiyan Hu, Qi Zhou, Yanbiao Jiang, Zhizhong Shang, Fan Mei, Qianqian Gao, Fei Chen, Li Zhao, Mengyao Jiang, Bin Ma

**Affiliations:** ^1^Evidence-Based Nursing Center, School of Nursing, Lanzhou University, Lanzhou 730000, China; ^2^The First Clinical Medical College, Lanzhou University, Lanzhou 730000, China; ^3^The Second Clinical Medical College, Lanzhou University, Lanzhou 730000, China; ^4^Evidence-Based Medicine Center, School of Basic Medical Sciences of Lanzhou University, Lanzhou 730000, China

## Abstract

**Objective:**

Chronological age alone does not adequately reflect the difference in health status of a patient with hypertension. Frailty is closely associated with biological age, and its assessment is clinically useful in addressing the heterogeneity of health status. The purpose of our study is to comprehensively examine the predictive value of frailty for negative health outcomes in hypertensive patients through a systematic review and meta-analysis.

**Methods:**

Multiple English and Chinese databases were searched from inception to 04.11.2020. All cross-sectional and longitudinal studies that examined the association between frailty and relevant clinical outcomes among hypertensive patients were included. The NOS was used to assess the risk of bias of studies included in the analysis. Hazard ratios (HRs), odds ratios (ORs), and 95% confidence intervals (CIs) were pooled for outcomes associated with frailty.

**Results:**

Six longitudinal studies and one cross-sectional study involving 17403 patients with hypertension were included in the meta-analysis. The risk of bias of all included studies was rated as low or moderate. The pooled HR of frailty related to mortality was 2.45 (95% CI: 2.08-2.88). The pooled HR of prefrailty and frailty-related injurious falls was 1.07 (95% CI: 0.83-1.37) and 1.89 (95% CI: 1.56-2.27), respectively. The pooled HR of prefrailty and frailty-related hospitalization was 1.54 (95% CI: 1.38-1.71) and 1.94 (95% CI: 1.17-3.24), respectively.

**Conclusions:**

This systematic review suggests that frailty was a strong predictor of mortality, hospitalization, and injurious falls among patients with hypertension. Our findings indicate that assessment of frailty in patients with hypertension to guide their management may be necessary in clinical setting. However, our finding was based on very limited amount studies; thus, future studies are required to further validate the role of frailty in prediction of negative health outcomes in hypertensive patients as well as pay more attention to the following knowledge gaps: (1) the association between frailty and hypertension-related outcomes, (2) the significance of the association between different frailty models and relevant clinical outcomes, and (3) the predictive value of prefrailty for the negative health outcomes in people with hypertension.

## 1. Introduction

Arterial hypertension is highly prevalent among older adults [1]. As a major risk factor for both cardiovascular and cerebrovascular diseases, hypertension is a pivotal factor for overall mortality risk in the older population [2, 3]. It also has a critical impact on quality of life of older people and the maintenance of their daily activities [2]. Antihypertensive treatment has been shown to reduce stroke, cardiovascular events, and mortality [4, 5]. However, the optimal treatment for older hypertensive patients remains controversial [6, 7].

In recent years, frailty has been the focus of much attention in the field of hypertension. Frailty is a biological state that results from a deterioration of multiple physiological systems, resulting in high vulnerability when an individual's health state changes [8, 9]. The population of older people with hypertension is highly heterogeneous, and even individuals of the same age are greatly variable in their physiological capability and vulnerability [10]. Chronological age alone does not adequately reflect the difference in health status of a patient with hypertension. Frailty, a reflection of decreased physiological reserve, is closely associated with biological age [11, 12], and its assessment is clinically useful in addressing the heterogeneity of health status among older people [13]. For older hypertensive patients with frailty, blood pressure management should differ to that used for the healthy older people, and that frailty should be considered when formulating the antihypertensive treatment plan [14–16].

The risk of frailty is associated with the presence of hypertension [17, 18]. The incidence of frailty in older people with hypertension has been revealed to be 32% (95% CI: 21%-43%) in meta-analyses [19]. As an effective indicator to objectively reflect the health status and medical needs of older people [13], the association between frailty and prognosis among patients with hypertension is worthy of examination. Moreover, given that frailty could be improved by appropriate intervention, it is considered that its early detection would be important for expeditious intervention [20]. Therefore, the objective of the present meta-analysis was to comprehensively examine the association between frailty and relevant clinical outcomes among patients with hypertension and provide evidence for the management of this group of the population in the future.

## 2. Methods

The study design and reporting of data of this systematic review are compliant with the Observational Studies in Epidemiology (MOOSE) guidelines [21] (Appendix S1). The review followed a predetermined, but unpublished protocol.

### 2.1. Search Strategy

Electronic Chinese and English-language databases, including PubMed, Embase, Web of Science, Cumulative Index of Nursing and Allied Health Literature (CINAHL), China National Knowledge Infrastructure (CNKI) database, Chinese Scientific Journals Full-Text Database, Wanfang database, and China Biological Medicine (CBM) database, were searched independently by two reviewers (K-YH and Y-BJ) from the inception of each to 04.11.2020. The following keywords were used: (“frail^∗^” OR “frailty”[Mesh] OR “frail elderly”[Mesh]) AND (“hyperten^∗^” OR “raised blood pressure” OR “high blood pressure” OR “hypertension”[Mesh] OR “subclinical cardiovascular disease” OR “cardiovascular risk factors”). The search strategy used in PubMed is displayed in Appendix S2. A similar search (adapted to the requirements of each database) was conducted of the other databases. References from each selected article were screened for potential additional studies.

### 2.2. Eligibility Criteria

*Study design*: quantitative studies of a cross-sectional or longitudinal design examined the association between frailty and relevant clinical outcomes in hypertensive patients. The publication language of articles was limited to either English or Chinese. Literature in which the original data could not be obtained, or a Newcastle Ottawa Scale (NOS) score < 5 was excluded.

*Participants*: these are patients with hypertension who were confirmed using clinical guideline thresholds recommended by any hypertension societies or were receiving antihypertensive drug treatment.

*Exposure*: frailty was defined as a state of vulnerability and measured at baseline through at least one established frailty model [22] (e.g., phenotype model [8], cumulative deficit model [23], or comprehensive geriatric assessment (CGA) [24]) or a modified version. The established frailty measure means the tool had published research data that verified its diagnostic accuracy.

*Outcome measures*: the primary outcomes were (1) all-cause mortality, (2) all-cause hospitalizations, and (3) injurious fall (defined as fall-related fractures, brain injuries, or joint dislocations requiring treatment). Planned secondary outcomes included are as follows: hypertension-related cardiovascular outcomes (e.g., myocardial infarction or stroke), hypertensive end-organ damage (e.g., renal function, plasma levels of B-type natriuretic peptide (BNP), and brain white matter hyperintensity (WMH)), and other markers of general morbidity (including quality of life, function, and independence).

### 2.3. Study Selection

All results were exported to the Endnote X8 software for the removal of duplicates. Two reviewers (K-YH and Z-ZS) independently scanned all titles and abstracts based on the eligibility criteria outlined above to identify potentially eligible studies. Prior to the formal scan, a random sample of 10% of records was independently scanned by the two reviewers. The complete scan did not commence until good agreement (>90%) was achieved between them. The two reviewers independently assessed the full texts of potential eligible studies on the basis of the stated eligibility criteria. Any disagreements between the two were resolved by discussion with a third (BM).

### 2.4. Data Extraction

Two reviewers (K-YH and QZ) independently extracted data from the selected eligible studies in a standardized, predefined Microsoft Excel spreadsheet. Extracted data were reviewed and cross-checked by the two reviewers prior to cleaning and analysis. Any disagreements between the two reviewers were resolved by discussion with a third (BM).

The following information was extracted: (1) first author and year of publication, (2) study design, (3) characteristics of the study population (e.g., sample/subsample size, percentage of women, age, and country), (4) setting in which the study was performed, (5) diagnostic criteria for frailty and identification of hypertension, (6) percentage of frailty in hypertension, (7) outcomes, (8) effect size (HRs, ORs, and 95% CIs), (9) adjustment factors, and (10) follow-up period. When several adjusted HRs and ORs were available in a study, the most adjusted estimate was extracted. For duplicated study populations, the one with the longest follow-up or the largest sample size was selected. Corresponding authors were contacted when it was not possible to extract the necessary data from a published paper.

### 2.5. Assessment of Risk of Bias

Quality of the studies was assessed independently by two reviewers (K-YH and QZ) using a tool for the qualitative evaluation of observational studies utilizing the NOS [25] which consists of 3 parameters of quality: selection, comparability, and exposure assessment. Studies scoring >7 were considered low risk of bias, scores of 5-7 indicated moderate risk of bias, and scores of <5 indicated high risk of bias. Any disagreement between the two reviewers in the quality assessment was resolved by discussion with a third (BM).

### 2.6. Statistical Analysis

Analyses were performed using the Revman 5.3 software. The pooled risk estimate of HRs, ORs, and 95% CIs extracted from the studies included in the review or calculated from the extracted data were examined to summarize outcomes associated with frailty. Statistical heterogeneity was measured using the *I*^2^ statistic. In the absence of statistical heterogeneity among the results, the meta-analysis was performed using the Mantel-Haenszel statistical method with the fixed-effects model. In the presence of statistical heterogeneity (defined as *p* < 0.10 and *I*^2^ > 50% [26]), subgroup analysis or sensitivity analysis was performed to identify its cause, and the Mantel-Haenszel statistical method with the random-effects model was used for meta-analysis. Planned subgroup analysis includes frailty state (frail/prefrail vs. robust), study setting (community vs. hospital), receipt of antihypertensive treatment, diagnostic criteria for frailty (phenotype model vs. cumulative deficit model vs. CGA vs. others), age (≥60 vs. <60 years), or sex (male vs. female). Publication bias was assessed using funnel plots if ≥10 studies were available [27]. *p* < 0.05 was considered statistically significant.

## 3. Results

**Figure 1:**
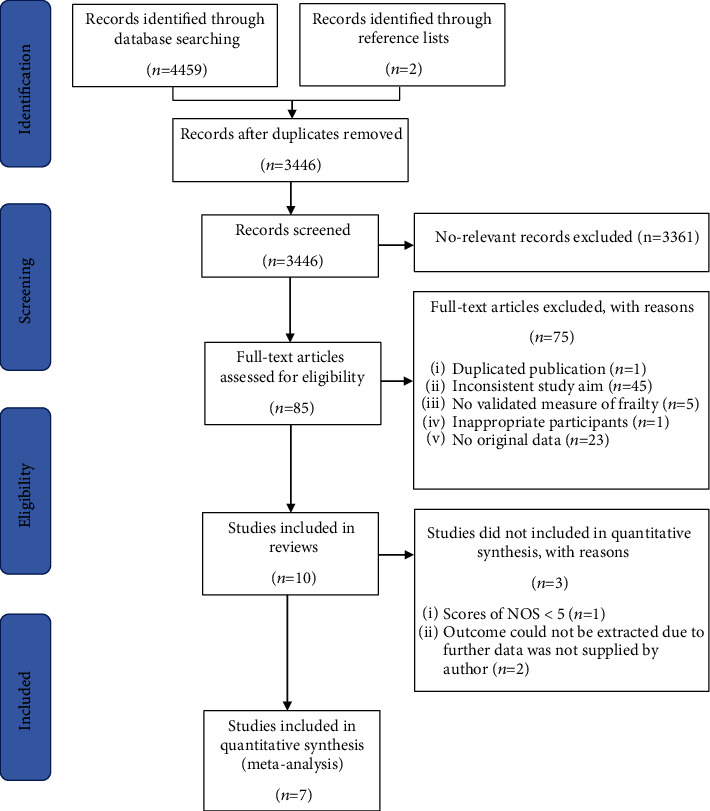
Flowchart describing the selection of studies.

The study selection process is presented in Figure 1. After detailed assessment based on eligibility criteria, 10 studies were included in the review and 7 were included in the meta-analysis [28–34] of which 2 [31, 33] required further data to extract outcomes from the Kaplan-Meier survival curves, which were supplied by the authors (Appendix S3). Since no reply was received from the authors of two studies [35, 36] after three attempts at contact and one study had a NOS score < 5 [37], the three had to be excluded from the meta-analysis.

### 3.1. Study and Participant Characteristics

The characteristics of the 7 studies (*n* = 17403 patients) are shown in Table 1. In 3 studies [28, 31, 33], the risk of bias was rated as low, and the remaining 4 studies [29, 30, 32, 34] were rated as moderate. Of the studies, 6 had a longitudinal design [28–33] and one was a cross-sectional study [34]. The follow-up period of the included longitudinal studies ranged from 0.4 to 11.9 years. In terms of frailty criteria, four studies [28, 32–34] used a phenotype model (or modified version) for defining frailty. One [29] used a cumulative deficit model, one [30] used the CGA, and one [31] used both a phenotype model and cumulative deficit model. The incidence of frailty was approximately 20.7%. For setting of the studies, four [28, 30, 33, 34] were conducted in the hospital and three [29, 31, 32] within community dwellers. For participants included in the studies, 42.1% were female (data unavailable from 3 studies [31, 33, 34]). In two studies [30, 34], middle aged and older people with hypertension were evaluated. Five studies [28, 30–33] researched only older people with hypertension.

### 3.2. Primary Outcome

#### 3.2.1. All-Cause Mortality

**Figure 2:**
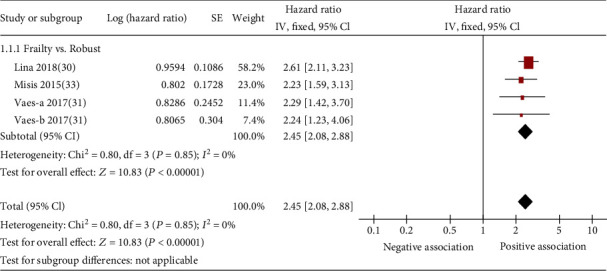
Forest plot of the association between frailty and all-cause mortality. Vaes-a 2017 describes a subsample of hypertension diagnosed by SBP of 140-160 mmHg; Vaes-b 2017 describes a subsample in which hypertension was diagnosed by SBP ≥ 160 mmHg.

On the basis of data from 3 cohort studies [30, 31, 33], the presence of frailty was significantly associated with increased hazard for all-cause mortality among patients with hypertension (pooled HR: 2.45; 95% CI: 2.08-2.88; Figure 2). One study reported data that it was adjusted appropriately. The median duration of follow-up for 1953 patients in the 3 studies was 5.30 years (quartile 1–quartile 3: 5.20-6.65 years). Since heterogeneity was low for frailty (*I*^2^ = 0%), a fixed-effects model was employed. No study examined the association between prefrailty and mortality among patients with hypertension.

#### 3.2.2. All-Cause Hospitalization

**Figure 3:**
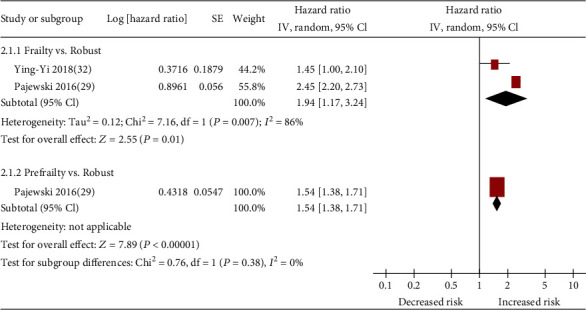
Forest plot of the association between frailty or prefrailty and all-cause hospitalization.

On the basis of data from 2 cohort studies [29, 32], the presence of frailty (pooled HR: 1.94; 95% CI: 1.17-3.24; Figure 3) or prefrailty (HR: 1.54; 95% CI: 1.38-1.71; Figure 3) was significantly associated with increased hazard of all-cause hospitalization among patients with hypertension. All studies reported data that were appropriately adjusted. The median duration of follow-up for 9654 patients in the 2 studies was 1.50 years (quartile 1–quartile 3: 0.95-2.05 years). Since heterogeneity was high for frailty (*I*^2^ = 86.0%), a random-effects model was employed.

#### 3.2.3. Injurious Falls

**Figure 4:**
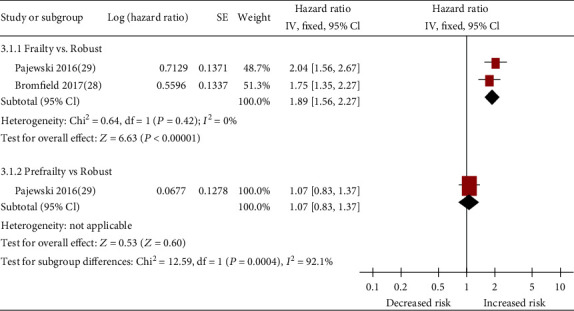
Forest plot of the association between frailty or prefrailty and injurious falls.

On the basis of data from 2 cohort studies [28, 29], the presence of frailty (pooled HR: 1.89; 95% CI: 1.56-2.27; Figure 4) was significantly associated with increased hazard for injurious falls among patients with hypertension. All studies reported data that were appropriately adjusted. The median duration of follow-up for 14542 patients in the 2 studies was 4.5 years (quartile 1–quartile 3: 3.55-5.45 years). Since heterogeneity was low for frailty (*I*^2^ = 0%), a fixed-effects model was employed. One study [29] reported prefrailty-related injurious falls. After appropriate adjustment, prefrailty was not significantly associated with high odds ratios for injurious falls (HR, 1.07; 95% CI, 0.83-1.37; Figure 4).

### 3.3. Secondary Outcome

**Figure 5:**
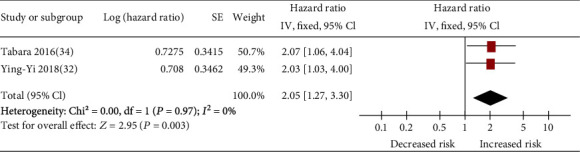
Forest plot of the association between frailty and kidney damage (presence of proteinuria) among hypertensive patients.

Two studies [32, 34] reported hypertensive end organ damage among 908 hypertensive patients. After appropriate adjustment, frailty was significantly associated with high odds ratios for kidney damage (presence of proteinuria: pooled OR 2.05 and 95% CI 1.27-3.30; Figure 5). Since heterogeneity was low for frailty (*I*^2^ = 0%), fixed-effects models were employed. One study [34] reported frailty-related damage in the brain (presence of WMH: OR 1.93 and 95% CI 1.08-3.44) and heart (presence of high BNP: OR 3.53 and 95% CI 1.44-8.66) and found a significant association. No studies examined the association between frailty and other prespecified secondary outcomes in patients with hypertension.

## 4. Discussion

In the present meta-analysis based on six cohort studies and one cross-sectional study, a significant association was observed between frailty status (frail>prefrail>robust) and high risk of injurious falls and all-cause hospitalization among patients with hypertension. Regarding mortality and hypertensive end organ damage, a signification association between frailty and these two outcomes was observed among patients with hypertension, but no study has at present examined the association between prefrailty and them.

Although the precise mechanisms underlying the association between frailty and subsequent higher mortality, injurious falls, and hospitalization among patients with hypertension remain unclear, we hypothesize that the following mechanisms can be considered. Previous studies have found that frailty was related to subclinical cardiovascular disease [38], cerebral small vessel disease [39, 40], or poor renal function [41]. Clinical phenotype of frailty is a symptom of subclinical organ damage [8]. The heart, brain, and kidneys are the principal target organs of hypertension. Long-term hypertension and poor control of blood pressure can lead to systemic arteriolopathy, leading to ischemia and damage to those organs [42], possibly increasing the detection of frailty. Hypertensive end organ damage could be a strong predictor of future mortality and hospitalization [43]. The association between frailty and hypertensive end organ damage among patients with hypertension was found in the present meta-analysis, also confirming this mechanism to a certain degree. Furthermore, the association between frailty and hospitalization among patients with hypertension could also be explained by injurious falls. Accidental falls were the mechanism linking frailty and hospitalization, which have been demonstrated in previous studies [44]. In the present meta-analysis, we observed a particularly robust association between frailty and injurious falls among patients with hypertension. Injurious falls may contribute even more to frailty-related hospitalization. Since the reason for hospitalization was investigated in only one included study [32], which involved cardiovascular events, cerebrovascular events, hypertension, infection, and falls, further examination of these mechanisms is necessary in the future. In addition, due to impairment of the regulatory mechanisms to preserve perfusion of vital organs in people with frailty, high blood pressure may be a compensatory mechanism to maintain organ perfusion [10]. Evidence from systematic review of observational studies demonstrates no mortality difference for older people with frailty whose SBP is <140 mmHg, compared to those with a SBP > 140 mmHg [45]. Therefore, the frail older people with hypertension might easily be overtreated when clinicians have no consideration of frailty [15, 46]. Low blood pressure values are related to syncope, falls, injuries, and fractures in older people, which have been repeatedly shown in previous studies [47–49]. Since participants who are administered antihypertensive drugs were eligible for inclusion in the meta-analysis, higher numbers of injurious falls and hospitalization might also be explained by the mechanism described above. However, this hypothesis needs to be further verified in future research.

Frail hypertensive patients are not only at higher risk of hypertension-related cardiovascular and cerebrovascular events, but also at higher risk of hypotension-related events (falls, injuries, and fractures) [10, 49], and hypotension-related events might be more common in real life. A recent analysis based on a large real-world database demonstrated a significant increase in hospitalization for hip fractures in older patients within 30 days of starting antihypertensive treatment [50]. Assessment of frailty in patients with hypertension to guide the management of blood pressure has been proposed in several clinical guidelines [14–16]. However, due to the lack of strong evidence [15, 51] or assessment methods, the ideal degree of blood pressure control in older people with hypertension remains inconclusive. Given frailty is a dynamic state [52] and could be improved or even reversed through nutrition [53], exercise [54, 55], and avoidance of low blood pressure [10, 56], its early detection and prompt intervention are important for reversing frailty status and improving prognosis. We believe it is necessary to explore the effects of interventional therapy on the prognosis of hypertensive patients with frailty in future research.

This is the first systematic review and meta-analysis of summarizing the current evidence on the association between a state of frailty and a variety of negative health outcomes in people with hypertension. A robust methodology, according to MOOSE guidelines, was employed, including conducting a comprehensive systematic review using eight electronic databases to ensure reliability and accuracy of results. Furthermore, the meta-analyses showed dose-response findings: higher degree of frailty status (frail>prefrail>robust) was associated with a higher risk of future hospitalization and injurious falls. However, certain limitations of this systematic review should be recognized. First, since small number of studies was eligible, the conclusions on all outcomes have been based only on two or three studies only (even though they included large samples). Second, the participants have quite differences across studies in age, gender, definition of hypertension, and treatment target. Although we planned to conduct subgroup analysis according these variables, it was not realized due to limited information. Moreover, effect size after necessary adjustments was not available in some studies. Third, due to various frailty models were identified in limited amount studies, it was impossible to perform a subgroup analysis on difference in the significance of the associations between frailty model and relevant clinical outcomes. Therefore, although an association was observed between frailty and negative health outcomes in hypertensive patients, we believe that future research is required to further validate the role of frailty in prediction of relevant clinical outcomes.

The findings of this systematic review are valuable for clinicians because they could screen frailty for older hypertensive patients and start appropriate interventions to prevent them from being suffered adverse events. Additionally, the finding of this review may help clinicians to stratify patients on the basis of their frailty levels to deliver targeted and more personalized treatment plans. This systematic review highlighted several gaps in the knowledge to be filled by future research. First, only two cross-sectional studies investigated the association between frailty and hypertensive end organ damage, but it was a shame there were no cardiovascular outcomes noted. It is maybe not so surprising that frailty predicts frailty-related outcomes in people with hypertension, but would have been interesting to know if it also predicted hypertension-related outcomes, like myocardial infarction or stroke. Second, although a variety of frailty measures could be used to assess frailty [22], it is unknown which measures are most suitable to detect hypertensive patient at high risk of mortality, hospitalization, and injurious falls. Moreover, it was unclear what factors or causes are involved directly and indirectly in the association between frailty and these negative health outcomes. Third, there were insufficient data focused on the predictive value of prefrailty for the negative health outcomes in people with hypertension. Given that hypertensive patients have a higher prevalence of prefrailty [19] and prefrailty is more likely to be reversed by appropriate intervention [20], data obtained in longitudinal studies that focus on prefrailty-related clinical outcome will be useful for risk stratification and prognosis improvement in hypertensive patients.

## 5. Conclusions

The present systematic review suggests that frailty was a strong predictor of mortality, hospitalization, and injurious falls among patients with hypertension. Our findings indicate that assessment of frailty in patients with hypertension to guide their management may be necessary in clinical setting. However, our finding was based on very limited amount studies; thus, future studies are required to further validate the role of frailty in prediction of negative health outcomes in hypertensive patients as well as pay more attention to the following knowledge gaps: (1) the association between frailty and hypertension-related outcomes, (2) the significance of the association between different frailty models and relevant clinical outcomes, and (3) the predictive value of prefrailty for the negative health outcomes in people with hypertension.

## Figures and Tables

**Table 1:** Basic characteristics of the included studies.

Study (year)	Study design	Country	Setting	Sample size	Age (years)	Female (%)	Identification for hypertension	Frailty measurements	Frailty (%)	Follow-up period (years)	Outcome	Effect measure	Adjustment factors	NOS
Bromfield (2017) [28]	Longitudinal	United States	Community	5236	≥65	53.7	Taking antihypertensive drugs	Indicators of frailty^#^	5.9	6.4	Injurious falls	HR	Age, sex, race, region of residence, education, income, cigarette smoking, statin use, osteoporosis use, benzodiazepines use, albumin to creatinine ratio, diabetes, history of heart disease, history of stroke, SBP, DBP, number of antihypertensive medication classes taken	8
Pajewski (2016) [29]	Longitudinal	United States	Hospital	9306	≥50	35.5	SBP within the range of 130–180, 130–170, 130–160, or 130–150 mmHg while being on no more than 0/1, 2, 3, or 4 antihypertensive drugs, respectively	36-item frailty index	27.6	2.6	Injurious falls All-cause hospitalizations	HR	Age, sex, race/ethnicity, education, alcohol consumption, and treatment arm	7
Lina (2018) [30]	Longitudinal	China	Community	1111	≥60	53.3	SBP ≥ 140 mmHg or DBP ≥ 90 mmHg or self-reported hypertension or receiving antihypertensive drugs	CGA	19.6	8.0	All-cause mortality	HR	Age, sex	7
Vaes (2017) [31]	Longitudinal	Belgium	Hospital	301^∗^	≥80	NA	SBP ≥ 140 mmHg	Groningen Frailty Indicator; frailty phenotype; frailty index	30.0	5.1	All-cause mortality Cardiovascular mortality	HR	NA	8
Ying-Yi (2018) [32]	Longitudinal	China	Hospital	348	≥65	10.0	SBP ≥ 140 mmHg or DBP ≥ 90 mmHg or receiving antihypertensive drugs	Frailty phenotype	38.1	0.4	All-cause hospitalizations Hypertensive end-organ damage (^a^presence of proteinuria)	HR, OR	Age, malnutrition, cognitive decline, polypharmacy, complication, orthostatic hypotension, proteinuria	7
Misis (2015) [33]	Longitudinal	Spain	Community	541^∗^	≥65	NA	SBP ≥ 140 mmHg	Walking speed < 0.8 m/s	39.0	5.3	All-cause mortality	HR	NA	8
Tabara (2016) [34]	Cross-sectional	Japan	Community	560	≥45	NA	SBP ≥ 140 mmHg or DBP ≥ 90 mmHg or taking antihypertensive drugs	Simple frailty score^#^	12.1	NA	Hypertensive end-organ damage (^a^presence of proteinuria, ^b^WMH, or ^c^high BNP)	OR	Age, sex	7

^#^The most severe group was frailty, whose data were compared with the nonfrailty group. ^∗^Subgroup participant samples were obtained by contacting the corresponding author. ^a^Proteinuria was defined as urinary protein/creatinine ratio ≥ 0.1 g/g creatinine or routine urine test showed positive urine protein. ^b^The presence of WMHs was defined as periventricular hyperintensity (PVH) grade ≥ 2 and/or deep subcortical white matter hyperintensity (DSWMH) grade ≥ 3 (PVH was classified into five grades: grade 0, absent or only a “rim”; grade 1, limited lesion-like “caps”; grade 2, irregular “halo”; grade 3, irregular margins and extension into the deep white matter; and grade 4, extension into the deep white matter and subcortical portion; DSWMH was also classified into five grades: grade 0, absent; grade 1, ≤3 mm small foci and regular margins; grade 2, ≥3 mm large foci; grade 3, diffusely confluent; grade 4, extensive changes in the white matter). ^c^High BNP was defined as BNP ≥ 100 pg/ml. CGA: comprehensive geriatric assessment; SBP: systolic blood pressure; DBP: diastolic blood pressure; HR: hazard ratio; OR: odds ratio; NA: not applicable; BNP: B-type natriuretic peptide; WMH: white matter hyperintensity.

## Data Availability

The study data supporting this systematic review and meta-analysis are from previously reported studies and datasets, which have been cited. The processed data are available in the article.

## Supplementary Materials

Supplementary Materials Appendix S1: MOOSE checklist for meta-analyses of observational studies. Appendix S2: PubMed search strategy. Appendix S3: method of all-cause mortality extraction from Kaplan-Meier survival curves
