# Supplementary material for: Association between Frailty and Mortality, Falls, and Hospitalization among Patients with Hypertension: A Systematic Review and Meta-Analysis
Source: Biomed Res Int. 2021 Jan 27;2021:2690296. doi: 10.1155/2021/2690296 (PMC7861941; doi:10.1155/2021/2690296)
Supplement: Supplementary Materials — Appendix S1: MOOSE checklist for meta-analyses of observational studies. Appendix S2: PubMed search strategy. Appendix S3: method of all-cause mortality extraction from Kaplan-Meier survival curves [file 2690296.f1.docx]

**Appendix S1: PubMed Search Strategy**

#1"cardiovascular risk factors[Title/Abstract]

#2 subclinical cardiovascular disease[Title/Abstract]

#3 raised blood pressure[Title/Abstract]

#4 high blood pressure[Title/Abstract]

#5 hyperten*[Title/Abstract]

#6 "Hypertension"[Mesh Terms]

#7 #1 OR #2 OR #3 OR #4 OR #5 OR #6

#8 frail*[Title/Abstract]

#9 "Frail Elderly"[Mesh Terms]

#10 "Frailty"[Mesh Terms]

#11 #12 OR #13 OR #14 OR #15

#12 #7 AND #11 Filters: Publication date to 2020/11/04; Humans; Chinese; English

**Appendix S2:** **MOOSE checklist for meta-analyses of observational studies**

| **Item no.** | **Recommendation** | **Reported on page no.** |
| --- | --- | --- |
| **REPORTING OF BACKGROUND SHOULD INCLUDE** | |  |
| 1 | Problem definition | 3-4 |
| 2 | Hypothesis statement | 4 |
| 3 | Description of study outcome(s) | 5 |
| 4 | Type of exposure or intervention used | 5 |
| 5 | Type of study designs used | 5 |
| 6 | Study population | 5 |
| **REPORTING OF SEARCH STRATEGY SHOULD INCLUDE** | | |
| 7 | Qualifications of searchers (e.g., librarians and investigators) | 4 |
| 8 | Search strategy, including time period included in the synthesis and keywords | 4, Appendix S2 |
| 9 | The effort to include all available studies, including contact with authors | 6, Appendix S3 |
| 10 | Databases and registries searched | 4 |
| 11 | The search software used, name and version, including special features used (e.g., explosion) | 4 |
| 12 | Use of hand searching (e.g., reference lists of obtained articles) | 5 |
| 13 | List of citations located and those excluded, including justification | 5 |
| 14 | Method of addressing articles published in languages other than English | 5 |
| 15 | Method of handling abstracts and unpublished studies | 5 |
| 16 | Description of any contact with authors | 6, Appendix S3 |
| **REPORTING OF METHODS SHOULD INCLUDE** | | |
| 17 | Description of relevance or appropriateness of studies assembled for assessing the hypothesis to be tested | 6 |
| 18 | The rationale for the selection and coding of data (discouraging, sound clinical principles, or convenience) | 6 |
| 19 | Documentation of how data were classified and coded (e.g., multiple raters, blinding and interrater reliability) | 6 |
| 20 | Assessment of confounding (e.g., comparability of cases and controls in studies where appropriate) | 7 |
| 21 | Assessment of study quality, including blinding of quality assessors, stratification, or regression on possible predictors of study results | 6-7 |
| 22 | Assessment of heterogeneity | 7 |
| 23 | Description of statistical methods (e.g., complete description of fixed or random effects models, justification of whether the chosen models account for predictors of study results, dose-response models, or cumulative meta-analysis) in sufficient detail to be replicated | 7 |
| 24 | Provision of appropriate tables and graphics | 7 |
| **REPORTING OF RESULTS SHOULD INCLUDE** | | |
| 25 | Graphic summarizing individual study estimates and the overall estimate | Figure 2-5 |
| 26 | A table giving descriptive information for each study included | Table 1 |
| 27 | Results of sensitivity testing (e.g., subgroup analysis) | Figure 3, Figure 4 |
| 28 | Indication of statistical uncertainty of findings | 12-14 |
| **REPORTING OF DISCUSSION SHOULD INCLUDE** | | |
| 29 | Quantitative assessment of bias (e.g., publication bias) | 17-19 |
| 30 | Justification for exclusion (e.g., exclusion of non-English language citations) | Figure 1, 18-19 |
| 31 | Assessment of quality of included studies | 18-19 |
| **REPORTING OF CONCLUSIONS SHOULD INCLUDE** | | |
| 32 | Conclusions of alternative explanations for observed results | 15-17 |
| 33 | Generalization of the conclusions (e.g., appropriate for the data presented within the domain of the literature review) | 18-19 |
| 34 | Guidelines for future research | 19 |
| 35 | Disclosure of funding source | 20 |

**Appendix S3: Method of All-cause mortality Extraction From Kaplan-Meier Survival Curves**

| Study | Data obtained from author | Method | Software | Calculated HR(95%CI) |
| --- | --- | --- | --- | --- |
| Vaes (2017) | \| SBP high \| Robust \| Frail \| \| --- \| --- \| --- \| \| 140-160 mmHg \| 133 \| 55 \| \| SBP high \| Robust \| Frail \| \| ⩾160 mmHg \| 77 \| 36 \| | Reference [1][2] | Engauge Digitizer | 2.29(1.48-3.87)^a^  2.24(1.23-4.05)^b^ |
| Misis (2015) | \| SBP high \| Fast walker (n=475) \| Slow walker (n=339) \| \| --- \| --- \| --- \| \| ⩾140 mmHg \| 332 (69.9%) \| 209 (61.7%) \| \| DBP high \| Fast walker (n=475) \| Slow walker (n=339) \| \| ⩾90 mmHg \| 98 (20.6) \| 59 (17.4) \| | Reference [1][2] | Engauge Digitizer | 2.23(1.59-3.13)^c^ |

^a^ was calculated using the subsample: SBP 140-160mmHg; ^b^ was calculated using the subsample: SBP ⩾160mmHg; ^c^ was calculated using the subsample: SBP ⩾140mmHg (we chose a larger subsample SBP high rather than DBP high).

Reference

[1] Guyot P, Ades AE, Ouwens MJ, Welton NJ. Enhanced secondary analysis of survival data: reconstructing the data from published Kaplan-Meier survival curves. BMC Med Res Methodol. 2012;12:9. Published 2012 Feb 1. doi:10.1186/1471-2288-12-9

[2] Tierney JF, Stewart LA, Ghersi D, Burdett S, Sydes MR. Practical methods for incorporating summary time-to-event data into meta-analysis. Trials. 2007;8:16. Published 2007 Jun 7. doi:10.1186/1745-6215-8-16
